# Oilseed Extracts from Local Markets as Promising Coagulant Agents for Milk from Various Mammalian Species

**DOI:** 10.3390/foods11142137

**Published:** 2022-07-19

**Authors:** Katia Liburdi, Sofia Cucci, Marco Esti

**Affiliations:** Department of Agricultural and Forestry Sciences (DAFNE), Tuscia University, Via San Camillo de Lellis snc, 01100 Viterbo, Italy; sofiacucci@outlook.it (S.C.); esti@unitus.it (M.E.)

**Keywords:** oilseed coagulants, bovine, buffalo, goat, sheep, crude extracts, milk-clotting properties, curd yield

## Abstract

The aim of this study was to identify novel milk coagulants to be used in cheesemaking. For this purpose, aqueous extracts from safflower (*Carthamus tinctorius*), sunflower (*Helianthus annuus*), flax (*Linum usitatissimum*) and sesame (*Sesamum indicum*) seeds were tested for their caseinolytic (CA) and milk coagulating properties (MCA) in skim milk at temperatures of 25, 37, 50, 65 and 80 °C. The seed oil samples with the highest temperature ranges in regard to coagulation efficiency were then tested in cow, buffalo, goat and sheep milks and the MCA and curd yield (CY) parameters were measured at different temperatures. Due to their high milk coagulation efficiency (CE) in all types of milk and at different temperatures, the sesame and sunflower seed extracts proved to be particularly interesting and their CY parameters were similar to those obtained with animal rennet. Moreover, our results confirm that oilseed coagulants are capable of coagulating milk and can also be considered as potential animal rennet substitutes. This study provides valuable insights into the development of potential vegetable coagulants that could be used for various production processes aimed at specific target consumers.

## 1. Introduction

For centuries, calf rennet has traditionally been used for coagulating milk in cheese making, which is mainly obtained from the abomasal mucosa of suckling calves [1]. Chymosin (E.C. 3.4.23.4), the enzyme found in calf rennet whose primary function is to coagulate milk, belongs to the aspartic protease family and can specifically cleave the peptide bond in Phe105-Met106 of κ-casein, which affects curd draining properties and curd moisture, thus producing cheeses with remarkable textural and flavor properties that cannot be achieved using other coagulants. However, the demand for coagulating enzymes started exceeding supply almost 50 years ago and today there is only enough calf rennet to produce 20–30% of the total cheese production volume [2]. Therefore, the global increase in cheese production and consumption and the increase in the price of calf rennet have underlined the need to find new milk coagulating enzymes that can satisfactorily substitute animal coagulants in cheese production. Today, recombinant bovine chymosin and microbial coagulant results have proved to be suitable alternatives and are both widely used in the dairy industry for cheese production. Plant-derived coagulants have recently received much attention as possible substitutes for calf rennet. However, several authors [3,4,5] claim that vegetable rennets are characterized by poor milk coagulation properties due to their strong, non-specific proteolytic activity. Extensive casein hydrolysis during cheese production may be associated with low cheese yield and flavor and texture defects [6,7]. However, various plant aspartic proteases, extracted from *Cynara cardunculus* and certain *Solanum* plants, possess optimal milk-clotting properties in cheese production [8]. The aqueous extract obtained from *C. cardunculus* is extensively used to produce a large variety of ovine cheeses in the Mediterranean basin, while plant-based coagulants obtained from *Solanum* spp. are used to manufacture commercial Sudanese and Mexican cheeses [9]. There is a global interest in using plant proteinases as milk coagulants since they are aqueous extracts that can be used for producing cheeses for lacto–ovo vegetarians and can also be used for manufacturing Kosher and Halal foods [10]. Therefore, the search for new potential plant-based milk-clotting plant enzymes continues in order to make them industrially viable and meet the rising global demand for diversified, high-quality cheese production.

Linen (*Linum usitatissimum*), safflower (*Carthamus tinctorius*), sesame (*Sesamum indicum*) and sunflower (*Helianthus annuus*) are widely distributed over different geographical areas, their seeds are edible and are used for producing cooking oil. Economically, oilseed crops are the most profitable cash crops to cultivate both in developed and developing countries due to their ability to grow in a wide range of pedo–climatic conditions.

Of the aforementioned oil seeds, only sunflower seeds have recently been studied to determine their milk *coagulation properties* (MCP) [11,12]. The earliest written reference to the safflower seed was made by Lucius Junius Columella in his treatise De Re Rustica (c. 50 bc): ‘… though it can also be coagulated with the flower of the wild thistle or the seeds of the safflower….’ [13]. Aspartic endopeptidases with strong proteolytic activity were extracted from sesame seeds [14] and a cysteine protease was isolated from the flaxseed extract for pharmaceutical applications [15]. As reported by Tan-Wilson and Wilson [16], it is evident that oilseeds generally contain various types of proteolytic enzymes that play essential roles in germination and maturation. These proteases break down storage proteins into free amino acids for biosynthesis and energy generation.

Apart from the effectiveness of using oilseed proteases in food and biotechnological processes, little is known regarding their application as coagulants in cheese-making. The present research aimed to explore the milk-*coagulation* properties of linen, safflower, sesame and sunflower crude plant-seed extracts on the curd yields obtained by coagulating milk of different animal origins (bovine, buffalo, goat and sheep) in terms of temperature dependence and quality. The commercial chymosin from animal source was used as reference.

## 2. Materials and Methods

### 2.1. Materials

#### 2.1.1. Seed Materials and Reagents

Casein from bovine milk, sodium phosphate monobasic (99–102%), sodium phosphate dibasic (>99.0%), trichloroacetic acid (>99.0%) and acetic acid (99.7%) were purchased from Sigma-Aldrich (Milano, Italy). Sodium hydroxide and sodium acetate were supplied by Carlo Erba Reagents (Milano, Italy). Skimmed milk powder was purchased from Graziano s.a.s (Cosenza, Italy).

The seed samples used in the experiment: linen (Lin, *Linum usitatissimum L.*), sesame (*Sesamum indicum L.*) and sunflower (*Helianthus annuus L.*) were purchased from a local market and safflower (*Carthamus tinctorius L.)* was produced and supplied by “Azienda agricola Biagini Elvio” (Valentano, VT, Italy). All of the seeds used in this study were edible and intended for human consumption whose marketing regulations are set out by the Directive 2002/55/EC as amended by Directive 2003/61/EC and by Regulation (EC) no. 1829/2003. Edible seeds are dried at 50 °C (±10) until they reach a 7–9% moisture content, in order to maintain their organoleptic and nutritive properties.

The calf rennet (CR) was purchased from Caglificio Clerici (175 IMCU, Como, Italy) and it was used as a reference.

#### 2.1.2. Milk Samples

The individual milk samples from bovine, buffalo, goat and sheep herds were collected from local farms based in the Lazio Region in October 2021. The milk samples were collected during the morning milking and transferred at 4 °C to the laboratory at Tuscia University, where all samples were thermized (55 °C for 15 s) and all chemical parameters were then determined (MilkoScan FT6000; Foss, Hillerød, Denmark).

### 2.2. Experimental Procedures

#### 2.2.1. Preparation of Aqueous Oilseed Extracts

The dried seeds of *C. tinctorius* (Saf), *S. indicum* (Ses), *L. usitatissimum* (Lin) and *H. annuus* (Sun) were ground with a laboratory mill until a fine flour was obtained. As reported in Table 1, different extraction rates were applied for each oilseed sample. The aqueous extracts were prepared by soaking the ground powder samples in acetate buffer (pH 5) containing 5% (*w*/*v*) NaCl. The aqueous mixtures were kept at 4 °C for 24 h under mild agitation and the samples were then filtered (No 42 Whatman paper) and centrifuged for 20 min at 5000 rpm (Megafuge 16R TX-200, Thermo Scientific™, Waltham, USA.) to obtain crude extracts.

#### 2.2.2. Protein Concentration Determination

The total protein content of each vegetable (Lin, Saf, Ses and Sun) and the CR sample was measured using the Bradford assay [17]. This method of analysis involves the use of calibration curve obtained from a series of BSA standard solutions (0.1–1.2mg/mL). When protein concentration was higher than 1.2 mg/mL, the sample was diluted, and the dilution factor was considered (Table 1). The absorbance was recorded at 595 nm by the use of a Shimadzu 1240 UV-visible spectrophotometer (Kyoto, Japan).

#### 2.2.3. Caseinolytic Activity

The caseinolytic activity (CA) of the four crude extracts (Lin, Saf, Ses and Sun) was determined as reported by Anusha et al. [18]. Overall, 0.25 mL of the crude extract was incubated with 0.25 mL of 1% (*w*/*v*) bovine casein in 0.05 M NaOH with 0.5 mL of sodium phosphate buffer at pH 6.5. The enzymatic reaction was conducted for 20 min at 25, 37, 50, 65 and 80 °C. The reaction was stopped by adding 0.5 mL of 15% (*w*/*v*) trichloroacetic acid. Subsequently, the mixture was centrifuged at 1500× *g* for 15 min, an aliquot of 0.5 mL of the supernatant was mixed with 7.5 mL of NaOH (0.5 M) and Folin Ciocalteau (1:2, *v*/*v*). The absorbance was recorded at 660 nm by the use of a Shimadzu 1240 UV-visible spectrophotometer (Kyoto, Japan). One unit of caseinolytic activity (CA) was defined as the amount of the protease required to release 1 μg of tyrosine under standard assay conditions.

The relative CA (R_CA_, %) was calculated by considering the optimum temperature required for coagulant activity as 100%.

#### 2.2.4. Milk-Clotting Activity

The milk-clotting activity (MCA) was evaluated for the Lin, Saf, Ses and Sun samples as reported by Anusha et al. [18] in milk samples made from powdered bovine, buffalo, goat and sheep milk. MCA was assayed by adding 3 mL of the crude extract to 10 mL of milk incubated at different temperatures (25, 37, 50, 65 and 80 °C). The assay was performed in triplicate and coagulation times under 40 min were considered positive and included in the data set for the effect of temperature on MCA. One unit of milk-clotting activity was defined as the quantity of protein required to coagulate 1 mL of milk in 40 min (2400 s) at the temperature evaluated:

$$\mathrm{MCA},U/\mathrm{mgBSA}=\frac{\left(\frac{2400}{T}*\;\frac{S}{E}\right)}{\mathrm{mgBSA}}$$

where T = time required for curd formation (seconds), S = volume of milk (mL) and E = volume of the coagulant (mL).

The MCA relative activity (R_MCA_, %) was calculated considering the optimum temperature required for coagulation as 100%.

#### 2.2.5. Curd Yield

At the end of the clotting phase, each glass tube was removed from the sample rack and the obtained curd was separated from the whey. Separation was performed at room temperature using a conical funnel containing a concave metallic net. The curd was gently pressed onto the net with a stainless-steel spatula and left to drain for 15 min. The whey was collected in a plastic tube placed underneath. The curd and whey obtained were weighed with a precision scale.

The curd yield (CY) traits were %CY_F_, %CY_D_, and %CY_W_, calculated as the ratios of the weight (g) of fresh curd, curd dry matter and the water retained in the curd, respectively, to the weight of the processed milk (g) and multiplied by 100 [19].

### 2.3. Data Analysis

The nonlinear regression was performed using “Prism 6 for Windows” software (version 6.03, GraphPad Software Inc., CA, USA) in order to assume that the R_CA_ and R_MCA_ (%) follow the Gaussian distribution.

The one-way analysis of variance (ANOVA) was executed using DSTAT meta-analytic software [20]. The data obtained were analyzed for statistical significance for testing the: (i) effects of the single factors (extraction rate) on the MCA; (ii) significant differences between the curd yield parameters (CY_F_, CY_S_ and CY_W_), at each temperature incubation, considering the independent factors (coagulant and milk types). Tukey’s comparison was used to verify the significance between groups. A *p*-value ≤ 0.05 was considered significant.

The results are reported as the means ± standard deviations.

## 3. Results and Discussion

### 3.1. Protein Content in Aqueous Oilseed Extracts

The protein concentration in the crude extracts was determined (Table 1), which generally decreased in all samples due to the large number of solid particles able to absorb aqueous solutions that consequently reduces the amount of crude extracts. In sunflower and sesame, only the extraction ratios of 0.1 and 0.2 g/L did not affect protein concentration, while the largest amounts of protein were observed in the Saf_0.1_, Sun_0.1_, Sun_0.2_ and Sun_0.3_ samples.

As reported by several authors, the protein content in quiescent seeds represents a fundamental nitrogen source which is essential for plant growth and development [15,16,21]. Although the Bradford assay cannot be used to identify enzymatic proteins in aqueous extracts, the presence of proteolytic enzymes in oil seeds has already been proven by various authors [14,22,23].

### 3.2. Caseinolytic Activity (CA) and Milk-Clotting Activity (MCA)

**Figure 1:**
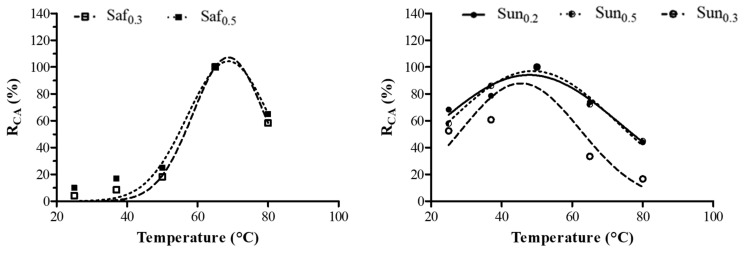
Effect of temperature on the caseinolytic activity (CA) of the crude safflower (Saf_0.3_ and Saf_0.5_) and sunflower (Sun_0.2_, Sun_0.5_ and Sun_0.3_) extracts obtained using different extraction rates (*w*/*v*): 0.2, 0.3, 0.5. The percentage (%) of the relative CA (R_CA_) represents the mean of three independent determinations performed in triplicate for each sample.

The effects of temperature on the CA of aqueous oil seeds extracts are reported in Figure 1. Only few safflower (Saf_0.3_ and Saf_0.5_) and sunflower (Sun_0.2_, Sun_0.3_ and Sun_0.5_) samples showed caseinolytic activity, with maximum CA temperatures at 65 °C and 50 °C, respectively. Hemalatha and Prasad [24] reported that protein degradation starts during oilseed germination and strictly depends on the type of protease stored in cell vacuoles. The proteolysis rate is highly variable, as the stored proteases catalyze different reactions. The lack of CA in the flax and sesame samples may be due to their non-specific casein hydrolysis which limits the release of tyrosine from micelles [3,4,13]. Indeed, chymosin, which has low proteolytic efficiency (data not shown), exhibited a high level of specificity for breaking the Phe105-Met106 peptide bond in k-casein. The excessive proteolytic activity of plant coagulants is the main limitation for their application in the dairy sector since the cheeses produced are characterized by low yields and flavor and consistency defects [11,25,26].

The crude linen, safflower, sesame and sunflower oil extracts were analyzed for their MCA in skim milk at different temperatures (Table 2). It is evident that the extraction ratio has a significant effect on the milk-clotting efficiency of the crude oilseed extracts. Among the linen and safflower samples, only Lin0.2 and Suf0.5 were found able to curdle the milk, while the sesame and sunflower extracts showed various MCA values for a wider range of extraction ratios. More specifically, for the crude sesame extracts, the MCA was measured from 50 °C when the solid/liquid ratio of 0.1, 0.2 and 0.3 g/L was used while the Ses0.5 proved to be effective from 37 to 80 °C. The sunflower oilseed samples also proved to be effective at the extraction rates of 0.1, 0.2 and 0.3 g/L and were already active at 37 °C.

Few studies compare the MCA from different vegetable sources and temperatures. However, Aworh and Muller [27] and Mazorra-Manzano et al. [8] obtained the same results using *Calostropis procera* and crude ginger extract as coagulating agents. The optimal temperature for MCA of vegetable coagulants depends on different factors, such as plant source, tissue, concentration and protease classes [28]. As expected, differences in MCA and protease activity (Table 1 and Table 2) were observed between the four oilseed extracts under study, which may be due to the proteases found in the aqueous extracts. The type of protease (e.g., cysteine, serine and aspartic) and its specificity is of great relevance and defines its affinity with the milk coagulation process. More specifically, cysteine proteases have a digestive proteolytic activity while aspartic proteases showed a specific for the Phe105–Met106 bond of bovine k-casein [29]. The present study, to our knowledge, is the first about proteolytic enzymes in oilseeds. However, the MCA observed cannot be attributed to any specific protease since they still need to be identified.

Nevertheless, promising results were obtained for the coagulating efficiency exhibited in the wide temperature range from 37–80 °C for the Lin_0.2_, Saf_0.5_, Ses_0.5_ and Sun_0.3_ samples. Therefore, these crude extracts were used in all of the experiments carried out on the milks of different animal origins.

### 3.3. Milk-Clotting Activity (MCA) and Curd Yield in Milk of Different Animal Species

**Figure 2:**
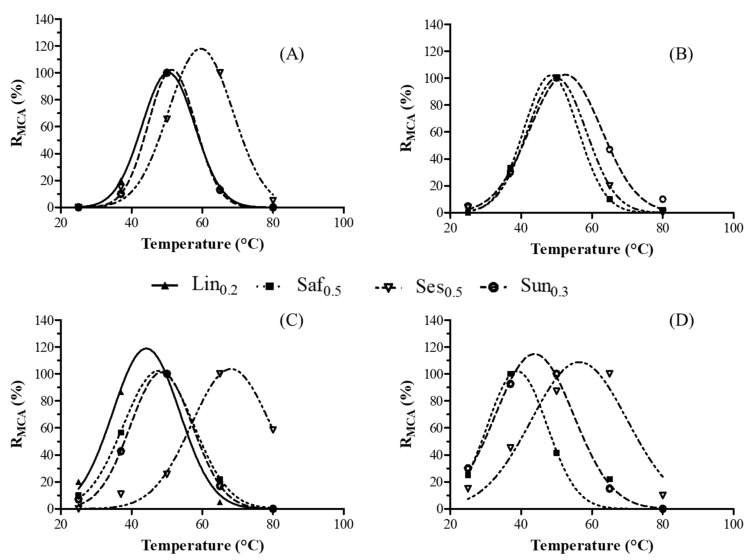
Effect of temperature on the milk-clotting activity (MCA) of the crude linen (Lin_0.2_), safflower (Saf_0.3_), sesame (Ses_0.5_) and sunflower (Sun_0.3_) extracts in bovine (**A**), buffalo (**B**), goat (**C**) and sheep (**D**) milks. The relative MCA (% R_MCA_) represents the mean of three independent determinations.

In the second section of the present study, the oilseed extracts were considered for their MCAs in bovine, buffalo, goat and sheep milks at different temperatures (Figure 2). The chemical compositions of the milks are reported in Table 3. The results show that all samples were of average-to-good quality with varying physical and chemical parameters [30,31].

The maximum MCA value for Lin_0.2_ was observed at approximately 50 °C and 40 °C in bovine and goat milk, respectively. Moreover, the coagulation activity of this crude extract was not observed in buffalo and sheep milk, which showed higher titratable acidity values (Table 3). This parameter plays an important role in all phases of milk coagulation, including the aggregation rate of para-casein micelles and coagulation reactivity [32]. The Saf_0.5_ sample exhibited maximal MCA values in buffalo and goat milk at temperatures between 40 °C and 60 °C while the 100% R_MCA_ was recorded at 37 °C when the same extract was added to sheep milk. No visible coagulation was observed at any temperature when bovine milk was incubated with Saf_0.5_, thus demonstrating that the milk coagulation efficiency of linen and safflower was affected by the type of milk. This is the first study conducted on using linen and safflower seeds as milk coagulants; therefore, there is no available scientific data with which to compare our results. However, it is well known that [33] the type of coagulant used is not the only factor affecting milk coagulation kinetics as there are several other influencing factors such as temperature, acidity, calcium and protein concentrations.

Conversely, oilseed extracts from sesame (Ses_0.5_) and sunflower (Sun_0.3_) showed clotting activity at different temperatures in all milk samples. More specifically, Ses_0.5_ reached the maximum MCA value at nearly 40 °C in buffalo milk and 60 °C in bovine, goat and sheep milk. Likewise, no previous studies have investigated the milk-clotting activity of sesame seeds. However, these findings are in agreement with a recent study on aspartic endopeptidases that were identified in sesame seed (SSE) aqueous extracts, which hydrolyze the proteins from bovine milk in the optimal temperature range of 40–70 °C [14]. Finally, as regards the Sun_0.3_ coagulant, the optimal temperature for milk-clotting activity is approximately 50 °C in all the milk samples. Several authors have identified the aspartic proteases from sunflower seeds that exhibited milk-clotting activity [11,12,34,35].

Milk coagulation can be divided into two phases: (a) the primary (or enzymic) phase, during which a proteolytic enzyme cleaves a peptide bond of casein creating a metastable state of the micelle, and (b) the secondary (or non-enzymic) phase when the milk subsequently gels and forms a clot. The primary phase exhibits the typical characteristics of an enzymatic reaction, such as pH and temperature dependence while temperature and milk composition affect the secondary phase of milk coagulation by controlling the hydrophobic interactions that occur during the aggregation of destabilized micelle. There is no apparent immediate reaction when the coagulating enzyme is added to the milk, then the milk appears to clot rapidly. However, if the aggregation of casein micelles and gel syneresis are prevented, no milk curdling occurs. Therefore, curd formation was not always observed in this study even if milk flocculation was observed in several samples. Although temperature had a significant effect on specific coagulation activity, it is equally important to measure the effect of the different vegetable coagulants on curd yield [4,36].

Since curd structure determines final cheese texture, the success in making cheese with the desired flavor and texture partly depends on its properties which determine the retention of fat and moisture and therefore cheese yield and composition. In this study, the milk curds were analyzed to determine the percentage of total curd weight (CY_F_) and dry (CY_D_) bases, and the water retained in the curd (CY_W_). The curd yield efficiency of the oilseed extracts was compared with the curd yield efficiency of commercial calf rennet.

As shown in Table 4, differences in CY_F_, CY_D_ and CY_W_ were significantly affected by temperature. Moreover, it was also observed that different milks responded differently to the various seed extracts. Despite the occurrence of the casein hydrolysis during the primary phase of the milk coagulation, a wide range of factors, such as micelle size, mineral content, the different casein to whey protein ratios and the complex interactions among them may hinder gel formation. An in-depth analysis of the effects of these characteristics on milk production using specific oilseed extracts is required. Our results clearly indicate the important roles that milk temperature and type play in determining curd characteristics. In this study, reducing the milk temperature led to increases in CY_F_, CY_D_ and CY_W_ parameters in curdled bovine, goat and sheep milks using vegetable and animal rennets. As reported by several authors [36,37,38,39,40], low milk temperature increases curd yield due to reduced syneresis, resulting in higher curd moisture contents, which may be due to curd shrinkage and increased wheying-off, which occurred during protein gelation at higher temperatures [36]. However, at temperatures between 50 to 25 °C in buffalo milk, comparable curd yields were obtained using both vegetable and animal rennets. Hussain et al. [41] observed a similar trend as a function of temperature for rennet gels demonstrating that curd moisture is temperature dependent, yet it is also affected by protein and fat contents. Moreover, a previous study by Stocco et al. [42] showed that milk samples rich in fat content have better milk coagulation properties, namely, shorter coagulation times, higher curd-firming rates, and earlier attainment of maximum curd firmness. Our results show that the highest curd yield values were obtained in buffalo and sheep milk, due to their high protein and fat content. In a study on cheese processing, Fox et al. [43] elucidated the relationships among the fat, casein and casein-to-fat ratios that are considered to be the main factors affecting cheese yield, as caseins create the continuous para-casein network occluding fat and water, but fat has limited water retention ability. In particular, increased milk fat concentration improved curd yield because it positively affected its protein and TS recovery rates. Fat also caused a higher water and total-solids retention in cheese. As reported by Pazzola et al. [19], milk protein improved curd nutrient recovery, especially of fat, which positively affected curd yield parameters.

Overall, significant differences were observed between the MCA and curd yield values of the oilseed extracts; however, their milk-clotting properties were demonstrated in real milk.

## 4. Conclusions

Safflower, sunflower, flax and sesame extracts have proved to possess coagulating properties in buffalo, goat, sheep and cow milk. However, their coagulation efficiency varies according to the methods and incubation temperatures used for extraction. For this reason, it is essential to select the most appropriate plant coagulant and type of protein gelation to enhance final product quality.

As expected, differences in milk-clotting and protease activities were observed between the evaluated extracts, which may be due to the different proteases predominant in each commercial oilseed.

In all types of milk heated at temperatures between 37 and 65 °C, cheese curd always formed. However, milk coagulation ability varied depending on the type of milk and the extraction methodology used. The results of this study confirm that the type of coagulant is not the only factor affecting milk coagulation kinetics, as there are several other influencing factors such as temperature and milk composition.

In conclusion, the results obtained from this study represent an important first step in identifying new seed coagulants for cheese production. Therefore, further research is required to evaluate the effects of other independent variables (i.e., pH, milk volume and calcium ion concentration) on oilseed coagulation efficiency. The implementation of an extractive protocol that guarantees the efficiency of new coagulants in terms of curd yield and consistency could confirm their suitability for various cheese production processes aimed at specific target consumers.

## Figures and Tables

**Table 1:** The protein content measured in the crude extract obtained from the four oilseed samples, linen (Lin), safflower (Saf), sesame (Ses) and sunflower (Sun) using different extraction rates (*w*/*v*, g/mL): 0.1, 0.2, 0.3, 0.5.

Enzyme Source	Extract Ratio (g/mL)	Acronymous	Protein Content (mgBSA/gr_DW_)
**Linen (Lin)**	0.1	Lin_0.1_	6.79 (±0.30)
	0.2	Lin_0.2_	4.34 (±0.27)
	0.3	Lin_0.3_	2.9 (±0.19)
	0.5	Lin_0.5_	2.25 (±0.1)
**Safflower (Saf)**	0.1	Saf_0.1_	2.75 (±0.64)
	0.2	Saf_0.2_	2.22 (±0.08)
	0.3	Saf_0.3_	2.55 (±0.310)
	0.5	Saf_0.5_	2.60 (±0.19)
**Sesame (Ses)**	0.1	Ses_0.1_	9.10 (±0.28)
	0.2	Ses_0.2_	6.48 (±0.02)
	0.3	Ses_0.3_	3.39 (±0.20)
	0.5	Ses_0.5_	3.01 (±0.19)
**Sunflower (Sun)**	0.1	Sun_0.1_	15.54 (±0.14)
	0.2	Sun_0.2_	16.25 (±0.70)
	0.3	Sun_0.3_	10.44 (±0.12)
	0.5	Sun_0.5_	7.08 (±0.01)

**Table 2:** Milk-clotting activity (MCA) measured in reconstituted skim milk powder at different temperatures (25°, 37°, 50°, 65° and 80° C), for the crude extracts obtained from linen (Lin0.1, Lin0.2, Lin0.3 and Lin0.5), safflower (Saf0.1, Saf0.2, Saf0.3 and Saf0.5), sesame (Ses0.1, Ses0.2, Ses0.3 and Ses0.5) and sunflower (Sun0.1, Sun0.2, Sun0.3 and Sun0.5) seeds at different extraction rates.

Enzyme Extracts	Temperature (°C)				
	**25°**	**37°**	**50°**	**65°**	**80°**
**Lin_0.1_**	n.d.	n.d.	2.26 ^f^ (±0.01)	n.d.	n.d.
**Lin_0.2_**	n.d.	2.3 (±0.03)	11.78 ^e^ (±0.35)	5.2 (±0.03)	n.d.
**Lin_0.3_**	n.d.	n.d.	n.d.	n.d.	n.d.
**Lin_0.5_**	n.d.	n.d.	n.d.	n.d.	n.d.
**Saf_0.1_**	n.d.	n.d.	n.d.	n.d.	n.d.
**Saf_0.2_**	n.d.	n.d.	n.d.	n.d.	n.d.
**Saf_0.3_**	n.d.	n.d.	n.d.	n.d.	n.d.
**Saf_0.5_**	n.d.	3.16 (±0.2)	13.07 (±0.13)	4.89 (±0.02)	4.10 (±0.03)
**Ses_0.1_**	n.d.	n.d.	2.93 ^h^ (±0.02)	4.64 ^k^ (±0.02)	n.d.
**Ses_0.2_**	n.d.	n.d.	4.71 ^f^ (±0.04)	7.47 ^j^ (±0.52)	3.49 ^x^ (±0.01)
**Ses_0.3_**	n.d.	n.d.	5.03 ^e^ (±0.07)	3.12 ^l^ (±0.03)	2.25 ^y^ (±0.03)
**Ses_0.5_**	n.d.	2.1 (±0.03)	4.18 ^g^ (±0.03)	7.56 ^j^ (±0.22)	3.82 ^w^ (±0.06)
**Sun_0.1_**	n.d.	n.d.	0.31 ^g^ (±0.02)	0.91 ^l^ (±0.008)	n.d.
**Sun_0.2_**	n.d.	n.d.	0.40 ^f^ (±0.08)	4.28 ^k^ (±0.09)	n.d.
**Sun_0.3_**	n.d.	2.92 (±0.02)	7.34 ^e^ (±0.10)	6.42 ^j^ (±0.10)	4.57 ^w^ (±0.04)
**Sun_0.5_**	n.d.	n.d.	n.d.	n.d.	n.d.

Different lowercase letters differ significantly (Tukey’s test, *p* ≤ 0.05), considering the MCA value at each extraction rate incubated at temperature of 37 °C (a, b, c, d), 50 °C (e, f, g, h), 65 °C (j, k, l, m), 80 °C (w, x, y, z); n.d. not detected (flocculation has not been visually detected).

**Table 3:** Chemical composition of bovine, buffalo, goat and sheep milk samples.

	Density (mg/mL)	pH	Water (%)	Total Acidity (°SH)	Lactic Acid (%)	Ash (%)	Total Protein (%)	Caseins (%)	Fatty Matter (%)	Lactose (%)	Dry Matter (%)
Bovine	1.028	6.71	87.3 (±0.4)	8.2 (±0.40)	0.18 (±0.01)	0.60 (±0.03)	3.71 (±0.31)	2.89 (±0.24)	4.1 (±0.19)	4.62 (±0.08)	13.53 (±0.1)
Buffalo	1.031	6.71	82.2 (±0.3)	9.4 (±0.30)	0.2 (±0.01)	0.88 (±0.02)	4.15 (±0.27)	3.71 (±0.21)	7.5 (±0.52)	4.74 (±0.2)	18.21 (±0.1)
Goat	1.028	6.65	87.2 (±0.4)	8.3 (±0.20)	0.18 (±0.01)	0.65 (±0.06)	3.74 (±0.16)	2.74 (0.12)	4.0 (±0.23)	4.51 (±0.1)	12.36 (±0.2)
Sheep	1.037	6.34	81.4 (±0.5)	11.3 (±0.10)	0.26 (±0.01)	0.82 (±0.05)	6.90 (±0.48)	5.59 (±0.38)	6.0 (±0.34)	4.45 (±0.1)	19.27 (±0.31)

**Table 4:** Curd yield parameters (CY_F_, CY_s_, CY_w_, %) of the seed extracts obtained from linen (Lin_0.2_), safflower (Saf_0.5_), sesame (Ses_0.5_) and sunflower (Sun_0.3_) in bovine, buffalo, goat and sheep milks at different incubation temperatures (25, 37, 50, 65 and 80 °C). Calf rennet (CR) was incubated under the same conditions and used as a control.

		Lin_0.2_			Saf_0.5_			Ses_0.5_			Sun_0.3_			CR		
		CY_F_	CY_S_	CY_W_	CY_F_	CY_S_	CY_W_	CY_F_	CY_S_	CY_W_	CY_F_	CY_S_	CY_W_	CY_F_	CY_S_	CY_W_
Milk	Temperature (°C)	%														
Bovine	25	NA	NA	NA	NA	NA	NA	NA	NA	NA	NA	NA	NA	89 (±4.37)	22 (±0.97)	67 (±3.80)
	37	NA	NA	NA	NA	NA	NA	NA	NA	NA	NA	NA	NA	66 (±2.75)	13 (±2.18)	53 (±2.78)
	50	42.1 ^ef^ (±3.20)	7.4 ^f^ (±0.90)	34.7 ^E^ (±2.50)	NA	NA	NA	47.8 ^e^ (±2.75)	7.9 ^ef^ (±0.51)	36.5 ^E^ (±2.91)	38.2 ^fg^ (±3.51)	8.1 ^ef^ (±0.54)	16.9 ^G^ (±1.73)	36 ^g^ (±2.67)	9 ^c^ (±1.01)	27 ^F^ (±1.66)
	65	NA	NA	NA	NA	NA	NA	35.8 (±2.46)	7.4 (±1.54)	28.4 (±2.92)	NA	NA	NA	NA	NA	NA
	80	NA	NA	NA	NA	NA	NA	NA	NA	NA	NA	NA	NA	NA	NA	NA
Buffalo	25	NA	NA	NA	NA	NA	NA	NA	NA	NA	NA	NA	NA	76.1 (±4.46)	16.4 (±1.23)	55.6 (±4.23)
	37	NA	NA	NA	65.8 ^b^ (±4.82)	13.6 ^b^ (±0.10)	53.0 ^AB^ (±2.05)	65.6 ^b^ (±4.85)	12.9 ^b^ (±0.55)	53.6 ^AB^ (±6.17)	62.4 ^b^ (±2.14)	13.0 ^b^ (±0.35)	49.5 ^B^ (±1.85)	73.9 ^a^ (±4.23)	16.1 ^a^ (±1.75)	57.7 ^A^ (±3.18)
	50	NA	NA	NA	64.9 ^ef^ (±5.47)	13.4 ^f^ (±0.10)	51.6 ^F^ (±1.7)	69.5 ^ef^ (±2.89)	13.9 ^f^ (±0.39)	55.5 ^EF^ (±0.72)	65.2 ^f^ (±3.03)	13.8 ^f^ (±0.29)	51.4 ^F^ (±2.21)	75.6 ^e^ (±5.01)	16.5 ^e^ (±1.26)	59.1 ^E^ (±4.35)
	65	NA	NA	NA	NA	NA	NA	NA	NA	NA	44.4 ^j^ (±4.47)	8.0 ^j^ (±0.77)	36.9 ^J^ (±1.98)	19.1 ^k^ (±0.25)	4.5 ^k^ (±0.17)	14.6 ^K^ (±5.49)
	80	NA	NA	NA	NA	NA	NA	NA	NA	NA	NA	NA	NA	NA	NA	NA
Goat	25	NA	NA	NA	NA	NA	NA	NA	NA	NA	NA	NA	NA	70.8 ^a^ (±2.75)	12.6 ^a^ (±0.51)	58.2 ^A^ (±4.35)
	37	40.1 ^b^ (±2.13)	7.5 ^a^ (±0.84)	29.1 ^A^ (±2.52)	39.7 ^a b^ (±0.66)	7.5 ^a^ (±1.15)	29.8 ^A^ (±0.78)	44.5 ^a b^ (±0.78)	7.8 ^a^ (±1.10)	33.4 ^A^ (±2.75)	45.1 ^a b^ (±2.43)	7.9 ^a^ (±1.19)	34.1 ^A^ (±1.71)	53.9 ^a^ (±2.51)	9.7 ^a^ (±0.70)	44.2 ^A^ (±4.01)
	50	22.5 ^f^ (±1.24)	6.0 ^e^ (±0.15)	16.6 ^EF^ (±1.38)	23.8 ^f^ (±1.85)	6.4 ^e^ (±0.62)	17.3 ^EF^ (±0.85)	24.4 ^f^ (±1.15)	6.4 ^e^ (±0.65)	18.0 ^EF^ (±1.16)	30.2 ^e^ (±2.75)	7.1 ^e^ (±1.34)	21.2 ^E^ (±2.80)	28.5 ^e^ (±0.24)	7.4 ^e^ (±0.65)	21.1 ^EF^ (±2.75)
	65	NA	NA	NA	NA	NA	NA	28.6 ^j^ (±1.15)	6.7 ^j^ (±0.83)	21.9 ^J^ (±2.61)	NA	NA	NA	20.2 ^k^ (±0.85)	6.3 ^j^ (±1.12)	13.9 ^K^ (±2.42)
	80	NA	NA	NA	NA	NA	NA	21.1 (±1.96)	4.3 (±0.52)	17.7 (±1.78)	NA	NA	NA	NA	NA	NA
Sheep	25	NA	NA	NA	NA	NA	NA	NA	NA	NA	NA	NA	NA	84.4 (±5.23)	23.1 (±1.70)	60.9 (±2.45)
	37	NA	NA	NA	75.5 ^a^ (±6.02)	13.9 ^b^ (±1.56)	60.3 ^AB^ (±5.79)	70.7 ^a^ (±5.23)	12.9 ^b^ (±1.34)	53.6 ^B^ (± 2.42)	80.7 ^a^ (±7.62)	15.1 ^b^ (±1.24)	65.6 ^A^ (±5.29)	77.3 ^a^ (±2.14)	17.5 ^a^ (±0.32)	59.7 ^AB^ (±2.09)
	50	NA	NA	NA	36.0 ^g^ (±2.11)	9.7 ^g^ (±1.02)	26.0 ^H^ (±1.95)	60.7 ^f^ (±3.24)	13.9 ^f^ (±0.71)	46.8 ^F^ (±1.24)	57.2 ^f^ (±3.25)	14.2 ^f^ (±1.24)	43.0 ^G^ (±1.44)	72.6 ^e^ (±2.54)	15.8 ^e^ (±0.21)	56.8 ^E^ (±1.23)
	65	NA	NA	NA	NA	NA	NA	40.2 (±2.64)	10.7 (±1.38)	29.5 (±2.54)	NA	NA	NA	NA	NA	NA
	80	NA	NA	NA	NA	NA	NA	NA	NA	NA	NA	NA	NA	NA	NA	NA

Results are mean values of triplicate experiments with standard deviations. The curd yield values with different letters differ significantly (Tukey’s test, *p* ≤ 0.05) considering the coagulant factor for the single milk animal source incubated at each temperature 37 °C (a-b-c-d, a-b-c-a-d and A-B-C-D for CY_F_, CY_S_ and CY_W_, respectively), 50 °C (e-f-g-h, e-f-g-h and E-F-G-H for CY_F_, CY_S_ and CY_W_, respectively), 65 °C (j-k-l-m, j-k-l-m and I-K-L-M for CY_F_, CY_S_ and CY_W_, respectively); NA: not applicable (the curd formation has not occurred).

## Data Availability

The date are available from the corresponding author.

## References

[1] Camin F., Bontempo L., Ziller L., Franceschi P., Molteni A., Corbella R., Verga I. (2019). Assessing the authenticity ofanimal rennet using δ 15 N analysis of chymosin. Food Chem..

[2] De Koning P.J. (1978). Coagulating enzymes in cheese making. Dairy Ind. Int..

[3] Ahmad S.M., Ahmad M.S., Amir Paray M. (2013). Plant proteases as milk-clotting enzymes in cheesemaking: A review. Dairy Sci. Technol..

[4] Liburdi K., Emiliani Spinelli S., Benucci I., Lombardelli C., Esti M. (2018). A preliminary study of continuous milk coagulation using Cynara cardunculus flower extract and calf rennet immobilized on magnetic particles. Food Chem..

[5] Liu X., Wu Y., Guan R., Jia G., Ma Y., Zhang Y. (2021). Advances in research on calf rennet substitutes and their effects on cheese quality. Food Res. Int..

[6] Nicosia F.D., Puglisi I., Pino A., Caggia C., Lucia Randazzo C. (2022). Plant Milk-Clotting Enzymes for Cheesemaking. Foods.

[7] Lo Piero A.R., Puglisi I., Petrone G. (2002). Characterization of “Lettucine”, a Serine-like Protease from *Lactuca sativa* Leaves, as a Novel Enzyme for Milk Clotting. J. Agric. Food Chem..

[8] Mazorra-Manzano M.A., Perea-Gutiérrez T.C., Lugo-Sánchez M.E., Ramirez-Suarez J.C., Torres-Llanez M.J., González-Córdova A.F., Vallejo-Cordoba B. (2013). Comparison of the milk-clotting properties of three plant extracts. Food Chem..

[9] González-Velázquez D.A., Mazorra-Manzano M.A., Martínez-Porchas M., Huerta-Ocampo J.A., Vallejo-Córdoba B., Mora-Cortes W.G., Moreno-Hernández J.M., Ramírez-Suarez J.C. (2021). Exploring the milk-clotting and proteolytic activities in different tissues of Vallesia glabra: A new source of plant proteolytic enzymes. Appl. Biochem. Biotechnol..

[10] Hashim M.M., Dong M., Iqbal M.F., Li W., Chen X. (2011). Ginger protease used as coagulant enhances the proteolysis and sensory quality of Peshawari cheese compared to calf rennet. Dairy Sci. Technol..

[11] Egito A.S., Girardet J.M., Laguna L.E., Poirson C., Mollé D., Miclo L., Humbert G., Gaillard J.L. (2007). Milk-clotting activity of enzyme extracts from sunflower and albizia seeds and specific hydrolysis of bovine κ-casein. Int. Dairy J..

[12] Nasr A.I.A.M., Mohamed Ahmed I.A., Hamid O.I.A. (2016). Characterization of partially purified milk-clotting enzyme from sunflower (*Helianthus annuus*) seeds. Food Sci. Nutr..

[13] Roseiro L.B., Barbosa M., Ames J.M., Wilbey R.A. (2003). Cheesemaking with vegetable coagulants-the use of Cynara L. for the production of ovine milk cheeses. Int. J. Dairy Technol..

[14] Chen Y., Zhu J., Zhang C., Kong X., Hua Y. (2021). Sesame water-soluble proteins fraction contains endopeptidases and exopeptidases with high activity: A natural source for plant proteases. Food Chem..

[15] Nandish S.K.M., Kengaiah J., Ramachandraiah C., Shivaiah A., Santhosh S.M., Sannaningaiah D. (2020). Flaxseed Cysteine Protease Exhibits Strong Anticoagulant, Antiplatelet, and Clot-Dissolving Properties. Biochemistry-Moscow.

[16] Tan-Wilson A.L., Wilson K.A. (2012). Mobilization of seed protein reserves. Physiol. Plant..

[17] Bradford M.M. (1976). A rapid and sensitive method for the quantitation of microgram quantities of protein utilizing the principle of protein-dye binding. Anal. Biochem..

[18] Anusha R., Singh M.K., Bindhu O.S. (2014). Characterization of potential milk coagulants from Calotropis gigantea plant parts and their hydrolytic pattern of bovine casein. Eur. Food Res. Technol..

[19] Pazzola M., Stocco G., Dettori M.L., Bittante G., Vacca G.M. (2019). Effect of goat milk composition on cheesemaking traits and daily cheese production. J. Dairy Sci..

[20] Johnson B.T. (1989). DSTAT: Software for the Meta-Analytic Review of Research Literatures.

[21] Staswick P.E. (1990). Novel Regulation of Vegetative Storage Protein Genes. Plant Cell.

[22] Gupta A., Sharma A. (2018). Solid state fermentation of non-edible oil seed cakes for production of proteases and cellulases and degradation of anti-nutritional factors. J. Food Biotechnol. Res..

[23] Müntz K. (1996). Proteases and proteolytic cleavage of storage proteins in developing and germinating dicotyledonous seeds. J. Exp. Bot..

[24] Hemalatha K.P.J., Prasad D.S. (2003). Changes in the metabolism of protein during germination of sesame (*Sesamum indicum* L.) seeds. Plant Foods Hum. Nutr..

[25] Esteves C.L.C., Lucey J.A., Pires E.M.V. (2002). Rheological properties of milk gels made with coagulants of plant origin and chymosin. Int. Dairy J..

[26] Jollès P., Alais C., Jollès J. (1963). Étude de la Caséine κ de vache caractérisation de la liaison sensible à l’action de la présure. Biochim. Biophys. Acta.

[27] Aworh O.C., Muller H.G. (1987). Cheese-making properties of vegetable rennet from sodom apple (*Calotropis procera*). Food Chem..

[28] Silvestre M.P.C., Carreira R.L., Silva M.R., Corgosinho F.C., Monteiro M.R.P., Morais H.A. (2012). Effect of pH and temperature on the activity of enzymatic extracts from pineapple peel. Food Bioprocess Technol..

[29] Liburdi K., Boselli C., Giangolini G., Amatiste S., Esti M. (2019). An evaluation of the clotting properties of three plant rennets in the milks of different animal species. Foods.

[30] Barłowska J., Szwajkowska M., Litwińczuk Z., Król J. (2011). Nutritional Value and Technological Suitability of Milk from Various Animal Species Used for Dairy Production. Compr. Rev. Food Sci. Food Saf..

[31] Boyazoglu J., Morand-Fehr P. (2001). Mediterranean dairy sheep and goat products and their quality: A critical review. Small Rumin. Res..

[32] De Marchi M., Fagan C.C., O’Donnell C.P., Cecchinato A., Dal Zotto R., Cassandro M., Penasa M., Bittante G. (2009). Prediction of coagulation properties, titratable acidity, and pH of bovine milk using mid-infrared spectroscopy. J. Dairy Sci..

[33] O’Callaghan D.J., Mulholland E.P., Duffy A.P., O’donnell C.P., Payne F.A. (2001). Evaluation of hot wire and optical sensors for on-line monitoring of curd firmness during milk coagulation. Ir. J. Agric. Food Res..

[34] Park H., Yamanaka N., Mikkonen A., Kusakabe I., Kobayashi H. (2000). Purification and Characterization of Aspartic Proteinase from Sunflower Seeds. Biosci. Biotechnol. Biochem..

[35] Walde P., Luisi P.L., Palmieri S. (1984). Proteolytic activity in sunflower seeds (*Helianthus annuus* L.). J. Agric. Food Chem..

[36] Fagan C.C., Castillo M., Payne F.A., O’Donnell C.P., O’Callaghan D.J. (2007). Effect of cutting time, temperature, and calcium on curd moisture, whey fat losses, and curd yield by response surface methodology. J. Dairy Sci..

[37] Everard C.D., O’Callaghan D.J., Mateo M.J., Castillo M., Payne F.A., O’Donnell C.P. (2011). Effects of milk composition, stir-out time, and pressing duration on curd moisture and yield. J. Dairy Sci..

[38] Johnson M.E., Chen C.M., Jaeggi J.J. (2001). Effect of rennet coagulation time on composition, yield, and quality of reduced-fat Cheddar cheese. J. Dairy Sci..

[39] Lucey J.A., Teo C.T.E.T., Munro P.A., Singh H. (1997). Rheological properties at small (dynamic) and large (yield) deformations of acid gels made from heated milk. J. Dairy Res..

[40] Riddell-Lawrence S., Hicks C.L. (1989). Effect of curd firmness on stirred curd cheese yield. J. Dairy Sci..

[41] Hussain I., Yan J., Grandison A.S., Bell A.E. (2012). Effects of gelation temperature on Mozzarella-type curd made from buffalo and cows’ milk: 2. Curd yield, overall quality and casein fractions. Food Chem..

[42] Stocco G., Pazzola M., Dettori M.L., Paschino P., Bittante G., Vacca G.M. (2018). Effect of composition on coagulation, curd firming, and syneresis of goat milk. J. Dairy Sci..

[43] Fox P.F., Guinee T.P., Cogan T.M., McSweeney P.L.H. (2017). Fundamentals of Cheese Science.

